# Topical steroid withdrawal: self-diagnosis, unconscious bias and social media

**DOI:** 10.1093/skinhd/vzaf051

**Published:** 2025-07-01

**Authors:** Jonathan Guckian, Olivia Hughes, Yasmin Nikookam, Ria Nair, Aqua Asif, Jeremy Brown, Anthony Bewley, Faheem Latheef

**Affiliations:** University of Leeds Institute for Medical Education, University of Leeds, Leeds, UK; School of Psychology, Cardiff University, Cardiff, Wales, UK; Internal Medicine Department, Guys and St Thomas Hospitals, London, UK; Newcastle University Medical School, Newcastle University, Newcastle upon Tyne, UK; Division of Surgery and Interventional Science, University College London, London, UK; Health Research Institute, Medical School, Faculty of Health, Social Care & Medicine, Edge Hill University, Ormskirk, Lancashire, UK; Department of Dermatology, Royal London Hospital, Bart’s Health NHS Trust, & Queen Mary University London, London, UK; Dermatology Department, Leeds Centre for Dermatology, Chapel Allerton, Leeds, UK

## Abstract

**Background:**

Consensus amongst dermatologists regarding the phenomenon of topical steroid withdrawal (TSW) is elusive. This may be contrasted with a growing online patient movement, including social media communities.

**Objectives:**

This study aimed to investigate dermatologist perspectives regarding TSW and to assess attitudes towards self-diagnosis.

**Methods:**

A two-part online questionnaire was disseminated to UK-based Dermatology Consultants, Registrars and Fellows. Section one presented a clinical scenario and randomized respondents into two groups: one mentioning TSW self-diagnosis, and an otherwise identical control without the self-diagnosis. Questions about the clinical scenario were directed to dermatologists and focused on attitudes regarding patient-predicted behaviours. Section two asked about TSW perceptions and experiences, and thematic analysis of open text responses was undertaken.

**Results:**

One hundred and three responses were received, including 51 Consultants, 38 Trainee Dermatologists, 10 Dermatology Fellows, 3 Specialty And Specialist (SAS) Dermatology doctors and 1 Post-CCT (Certificate of Completion of Training) Fellow. Thirty-four percent (*n* = 35/103) of respondents considered TSW to be a distinct clinical entity, 17.5% (*n* = 18/103) did not and 48.5% (*n* = 50/103) were unsure. Respondents felt that self-diagnosing TSW patients were less likely to comply with treatment, and more likely to take up time and pose management problems compared with controls. Themes of uncertainty regarding diagnostic veracity and social media misinformation were identified.

**Conclusions:**

Uncertainty regarding the veracity of a TSW diagnosis and its management is common amongst dermatology healthcare professionals (HCPs). Dermatology HCPs in this study considered that patients who self-diagnosed TSW were more difficult to engage with skin disease management. Dermatologists desire further understanding of and research into the nature and management of TSW.

## Teaser

This survey study amongst dermatology registrars and consultants has identified that many dermatologists encounter patients presenting with TSW self-diagnoses but uncertainty regarding its validity remains common. Clinicians see this phenomenon as strongly linked to eczema and as being social-media driven. Our study indicates that patients self-diagnosing with TSW were more likely to be viewed negatively in terms of treatment concordance and difficulty with management.

## Graphical Abstract

**Graphical Abstract:**
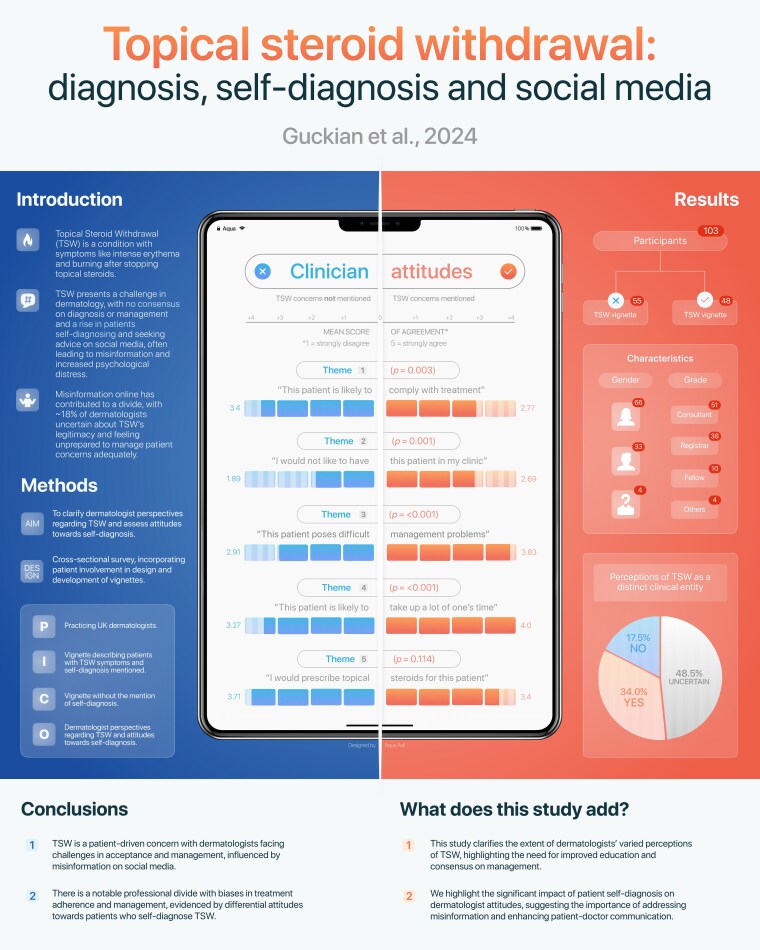

**What is already known about this topic?**

- Topical steroid withdrawal (TSW) has been reported as a reaction to the withdrawal of topical steroids, leading to skin redness and burning.
- Many dermatologists feel ill-equipped to manage patient expectations or do not consider it as a distinct clinical entity.
- TSW patients seeking support from inaccurate social media sources could be at increased risk of misinformation.

**What does this study add?**

- Dermatology HCPs have variable acceptance of the validity of TSW as a disease entity and of its management.
- A small minority (18%) of dermatologists believe TSW patients are receiving adequate care, but the majority do not feel confident about diagnosing TSW.
- Dermatologists perceive that unevidenced misinformation on social media has driven uncertainty relating to TSW, and believe that further educational and research support from institutions would help.

Topical steroid withdrawal (TSW) is not fully accepted as a specific clinical entity by some dermatology healthcare professionals (HCPs). Some dermatology HCPs believe that it is rebound eczema from cessation or inadequate use of effective topical cutaneous corticosteroid anti-inflammatories. However, patients with TSW describe an ‘intense erythema and burning which is more severe and further prolonged than can be explained by the rebound vasodilation that occurs after discontinuing topical corticosteroids’ (TCS).^1^ Acceptance and consensus around the veracity of a TSW diagnosis has not been achieved by dermatologists. This is exacerbated by the lack of clarity around the exact terminology of TSW, with terms such as ‘topical steroid addiction’ or ‘corticophobia’ being used.^2^ In addition, the symptoms and signs of TSW – erythema, scale, swelling, burning and stinging, oedema, mood disturbance, hair loss and skin pain – are not specific to TSW and may been seen in patients with rebound atopic eczema.^3–5^

The uncertainty surrounding TSW is reinforced by the lack of empirical evidence.^3,6,7^ As a result, the British Association of Dermatologists (BAD) and National Eczema Association have issued a position statement attempting to improve clarity about TSW terminology.^8^ In-depth understanding of dermatologist perceptions of TSW, or attitude towards self-diagnosis of TSW, is currently lacking. In this context, patient-led organizations such as the International Topical Steroid Awareness Network (ITSAN) and Scratch That have called for further research.^9,10^

Clinical uncertainty contrasts with a growing patient movement online, emphasizing a patient–dermatologist divide. Some HCPs believe TSW to be a ‘social media driven’ pathology, especially as there have been over 572 million views of #topicalsteroidwithdrawal on TikTok and a 274% increase in social media mentions of #topicalsteroidwithdrawal between 2016 and 2020.^11^ Patients, suspicious of dermatologist dismissal, seek support on diagnosis and management through active social media communities where doctors are conspicuous by their absence. The divide is being played out on the pages of academic journals, with patients reacting with distress to warnings surrounding TSW ‘misinformation’ and the description of TSW as ‘myth’.^12^ Mistrust of medical professionals has been reported amongst TSW patients, with breakdown of relationships due to perceived lack of empathy or acceptance of the condition highlighted as reasons for negative health-seeking behaviours such as self-discharge from dermatology services.^13^ However, a recent survey study by Barlow *et al*. highlighted that online awareness and patient perceptions may be beginning to influence dermatology practice.^14^

This is not the first divergence in perceptions of novel medical phenomena between doctors and patients. The long history and recent re-emergence of vaccine hesitancy indicates that trust between physicians and patients can be delicate.^15^ Disparities between patients’ and clinicians’ understanding may have implications on patient psychological wellbeing, and engagement with services.^16^ From the physician perspective, exposure to self-reported diagnosis of previous emergent, ‘unexplained’ conditions like myalgic encephalomyelitis (ME) or chronic fatigue syndrome (CFS) has historically led to negative clinician attitudes regarding patient beliefs, potentially influencing outcomes.^17^

Currently, further research is needed to delineate the awareness, understanding and practice of HCPs who may see patients with TSW. Therefore, this study aimed to determine dermatologist’s perspectives regarding TSW and investigate existing attitudes towards self-diagnosis.

## Methods

Our study used a cross-sectional survey to gather data from practising UK dermatology doctors. An online questionnaire was constructed using Qualtrics,^18^ featuring two distinct sections.^19,20^

In section one, respondents were randomized into two groups and presented with one of two case scenarios. The case scenarios were identical, but one mentioned self-diagnosis of TSW, and the other did not. Both scenarios featured a fictional case of a 28-year-old human resources officer experiencing TSW symptoms derived from the Medicines and Healthcare products Regulatory Agency (MHRA) definition of TSW.^21^ The scenarios also featured anonymized photographs derived from Shutterstock, a provider of open licence images using standard license.^22^ Photos were verified by a consultant dermatologist. In scenario one, the patient reported concerns about TSW, derived from their research on social media. In scenario two, this information was not provided. Subsequent questions focused on attitudes regarding patient predicted behaviours.

**Figure 1:**
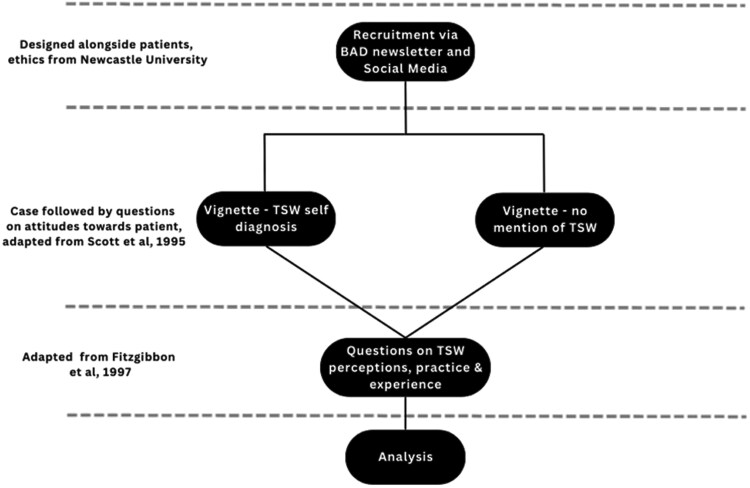
Methodological approach to this questionnaire study.

Section two separately aimed to investigate dermatologist acceptance of TSW and clarify exposure and current practice. Therefore, we adapted an established questionnaire from previous research on ME/CFS,^20^ as this condition has historically been associated with negative attitudes from HCPs. Therefore, adapting an established tool for TSW, a similarly emerging, under-reported phenomena, associated with negative discourse, provides a model for studying clinician perceptions and experiences. Given the established relevance of social media to TSW self-diagnosis, we also added a question on social media use to our questionnaire.^23^ Comparative data were analysed using IBM SPSS Statistics.^24^ Reflexive thematic analysis of qualitative data derived from long-form responses was undertaken^25^ (Figure 1). Questionnaire adverts and invitations were disseminated via social media (e.g. Instagram, X) and one circular via the BAD Newsletter, sent to all BAD members.

The study received ethical approval from Newcastle University (24440/2022) and participants granted informed consent prior to commencement of the questionnaire. Patient involvement in the design, data collection and analysis was via two patients with experience of living with a skin condition and TSW (O.H. and J.B.).

## Results

### Participant demographics

One hundred and three respondents to the survey met the inclusion criteria for the study including Dermatology Consultants (*n* = 51/103, 49.5%), Dermatology Registrars (*n* = 38/103, 36.9%), Dermatology Fellows (*n* = 10/103, 9.8%), Specialty and Specialist (SAS) Dermatology doctors (*n* = 3/103, 2.9%) and Post-CCT (Certificate of Completion of Training) Fellows (1/103, 1%). The study received responses from across the UK, including England (*n* = 78, 75.7%), Wales (*n* = 6, 5.8%), Scotland (*n* = 11, 10.7%) and Northern Ireland (*n* = 8, 7.7%). Of the participants, 66 were female (64%), 33 were male (32%) and 4 (3.8%) preferred not to disclose their gender.

### Scenario responses

Forty-eight (46.6%) participants were randomized to scenario one (TSW concerns mentioned from social media use) and 55 (53.4%) participants received scenario two (no TSW concerns mentioned). Differences in responses are outlined in Table 1. Like Scott *et al*.’s^20^ ME study, level of agreement was indicated on a five-point Likert scale and responses to scenarios were compared by the Mann–Whitney U test.

**Table 1:** Clinician attitudes towards TSW self-diagnosis vs without self-diagnosis

	Level of agreement: mean score (95% CI and SD)		
	Scenario one, TSW concerns mentioned	Scenario two, no TSW concerns mentioned	*P*-value (*n* = 103)
This patient is likely to comply with treatment	2.77 (CI: 2.46–3.07, SD: 1.06)	3.4 (CI 3.12–3.68, SD: 1.04)	0.003*
I would not like to have this patient in my clinic	2.69 (CI: 2.31–3.06, SD: 1.29)	1.89 (CI: 1.63–2.15, SD: 0.96)	0.001*
This patient poses difficult management problems	3.83 (CI: 3.60–4.07, SD: 0.81)	2.91 (CI: 2.6–3.22, SD: 1.14)	<0.001*
This patient is likely to take up a lot of one’s time	4 (3.78–4.22, SD: 0.74)	3.27 (CI: 3.07–3.68, SD 1.06)	<0.001*
I would prescribe topical steroids for this patient	3.4 (CI: 3.34–3.95, SD: 1.01)	3.71 (CI: 3.42–4.00, SD: 1.07)	0.114

Note: 1 = strongly disagree, 2 = disagree, 3 = neither agree nor disagree, 4 = agree, 5 = strongly agree. CI, confidence interval. *Significance at *P* < 0.05.

We found that the 46.6% (*n* = 48/103) of respondents who were allocated to the TSW self-diagnosis scenario were significantly more likely to believe that the patient was ‘more likely to pose difficult management problems’ (mean score 3.83/5 vs 2.81/5, *P* = 0.001) and ‘more likely to take up one’s time’ (4/5 vs 3.27/5, *P* < 0.001) than the 53.4% (*n* = 55/103) of participants allocated to the patient not self-diagnosing TSW. This 46.6% of respondents who were allocated to the TSW self-diagnosis scenario were also significantly more likely to state they would not like to have the patient in their clinic (2.69/5 vs 1.89/5, *P* = 0.001), and less likely to state that ‘the patient is likely to comply with treatment’ (2.77/5 vs 3.4/5, *P* = 0.003) compared with the 53.4% of respondents allocated to the patient not self-diagnosing TSW. There was no significant difference between these participant groups concerning whether they would prescribe TCS for their patient, indicating that self-diagnosis of TSW was not a factor for clinicians when considering prescribing TCS.

### Perspectives on TSW

**Figure 2:**
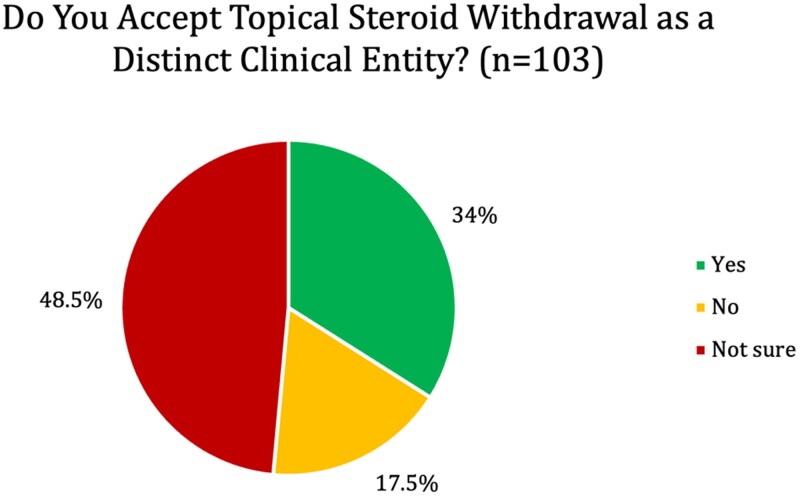
Participant perceptions of TSW as a distinct clinical entity.

Participants’ perspectives towards TSW as a diagnosis were assessed in section two. Respondents were asked about whether they felt TSW was a distinct clinical entity (Figure 2).

Confidence levels regarding diagnosis amongst clinicians varied. Of the participants, 52.3% (46 of the 88 who responded to this question) did not feel confident when it came to diagnosing TSW. Only 18% (*n* = 16/89) participants felt their TSW patients were receiving adequate care, whilst 37.5% (*n* = 33/89) felt that their TSW patients’ care was inadequate and 44.9% (*n* = 40/89) were unsure.

Concerning management, participants were able to choose more than one option. Clinicians felt ‘switching to emollients and relevant UV, systemic, or biologic therapy’ was the preferred treatment choice, with 78.6% (*n* = 81/103) of participants choosing this option. We identified that 39.8% (*n* = 41/103) chose to persist with ‘further topical steroids’, 24.3% (*n* = 25/103) chose to ‘switch to oral prednisolone’, whilst 24.3% (*n* = 25/103) chose to ‘cease topical steroids’. Finally, 22.3% (*n* = 23/103) wished to initiate ‘referral to psychology/psychodermatology/psychiatry services’ and 12.6% (*n* = 13/103) wished to switch to emollients only. The study also asked doctors to choose the conditions that they most see associated with TSW, with multiple responses possible. Eczema was the most popular choice with 97% participants (*n* = 100/103) selecting eczema and 4.9% choosing psoriasis (*n* = 5/103). No other conditions were chosen.

Clinicians were asked whether their patients stated their source of self-diagnosis of TSW. Social media was the most prevalent (82.5%, *n* = 85/103), followed by internet searches (68.9%, *n* = 71/103) and patients’ relatives and friends (35%, *n* = 36/103). Medical professionals were less prevalent as sources, with 6.8% (*n* = 7/103) identifying the patient’s GP as a source and 2.9% (*n* = 3/103) citing dermatologists as a diagnostic source.

The social media presence of clinicians themselves was studied, with all participants being asked to choose all the social media platforms on which they were active. Instagram (51.4%, *n* = 53/103) was the most popular platform, with Facebook (40.8%, *n* = 42/103) and LinkedIn (29.1%, *n* = 30/103) also proving popular.

### Qualitative themes

Qualitative data were gathered regarding perceptions on TSW, and reflexive thematic analysis was undertaken through open coding of responses.^25^ Three themes were identified from this analysis.

The theme of diagnostic uncertainty was identified, with several clinicians detailing that they did not feel, or could not be sure, that TSW was a distinct clinical entity. Participants reported a need for ‘a lot more research to establish diagnostic criteria and management guidance’, linking this to diagnostic difficulties in ‘differentiating escalation of disease with steroid withdrawal’. A profound ‘lack of teaching’ was noted by participants on this issue, but ‘support groups’ were cited as being helpful in filling educational gaps. One participant argued that responsibility lay with institutions, highlighting that they ‘would be more confident if there was published research on this or firmer statements from BAD/AAD etc.’

The most dominant theme related to misinformation. ‘A large bag of ailments being labelled as TSW’ was cited by dermatologists, and research was identified as a key step to ‘tackle disinformation about it… to reduce patient harm’. Strategies to identify potential risks of misinformation, or misinformed patients were shared, including ‘… a clue to knowing the information you are getting is false is criticism of doctors. Doctors have no interest in making your health worse…’. Concerns of patients that doctors are ‘paid by Big Pharma to prescribe’ were reported. Patients were also highlighted as being vulnerable to nonevidence-based practices driven by such misinformation, including ‘no moisture regimes’ and ‘alternative therapies which the evidence does not support’, whilst misinformation was blamed for patients ‘being ready for war’ and reminding clinicians of ‘vaccine deniers and COVID deniers’.

A third theme, linking those above, concerned social media. This was frequently claimed to be a driver of TSW self-diagnosis concerns and the host of more harmful behaviours or beliefs. Social media was argued to be ‘hyping this up’, with one respondent stating, ‘I have seen a lot more on Instagram than in clinical practice!’ Management options were perceived to be impacted by online behaviours and fixed beliefs, where a ‘straightforward approach (alternative agents) is often hindered by hype and distrust fuelled by social media’. This was linked to an absence of clinician social media presence, with beliefs felt to be drawn from ‘places on there without many doctors present’.

## Discussion

This study has identified that there is uncertainty about the validity and veracity of the diagnosis of TSW amongst dermatologists. Clinicians believe TSW to be linked to atopic eczema and to be social-media driven. Our study indicates that patients self-diagnosing with TSW were more likely to be viewed negatively in terms of treatment concordance and to be viewed as potentially difficult to engage. Only a minority of dermatologists (18%) in this study felt their TSW patients were receiving adequate care.

A recent questionnaire by Barlow *et al*. found that 96% of dermatologist respondents felt ‘most people complaining of TSW are simply experiencing ordinary eczema which relapsed because the TCS was stopped’.^14^ This contrasts with our cohort, where 34% believed that TSW was a distinct clinical entity. Whilst Barlow *et al*. found that 38.5% of respondents expressed a lack of confidence in TSW management, 52.3% of our participants did not feel confident diagnosing TSW. We also share the theme of lack of education and research driving uncertainty, though our study further highlights clinicians’ views that misinformation and social media are at the heart of TSW beliefs.

The most noteworthy findings in our study relate to negative attitudes clinicians hold towards patients presenting with TSW self-diagnosis. Patient perceived dismissal by HCPs regarding TSW has been frequently reported.^4,7,26^ One qualitative study linked negative clinician attitudes to patients withdrawing from care or seeking complementary therapies.^13^ However, our study is unique in highlighting that such negative attitudes may often be subconscious or subtle rather than relating to overt dismissal.

Unconscious biases in healthcare can reinforce racial, gender or other societal inequalities. However, specifically amongst physicians, it has been highlighted that uncertainty and time-pressures may lead to reliance on ‘medical stereotypes’, despite the best intentions.^27^ Prime examples of medically constructed stereotypes exist within fibromyalgia and CFS and are consistently framed in gendered contexts (particularly towards women), and may contribute to harmful scepticism regarding their diagnostic validity on the part of clinicians.^28^ Unconscious bias has been linked to uncertainty, a key theme from our study. Under pressured environments – a concern for our cohort – efforts to prematurely seek certainty in uncertain environments may allow implicit biases to hold more weight.^29^

To the best of our knowledge, this study is the first to suggest that social media, and subsequent perceived misinformation, may act as a driver for dermatologists’ unconscious biases. Social media has frequently been used as a lens to explore TSW. One study undertook analysis of social media/blog posts to investigate TSW patient health-seeking behaviours, identifying online searches and social media communities as playing an important role in self-diagnosis.^30^ Dermatologists’ professional use of social media has been investigated, with one review outlining its immense potential for patient education and challenging misinformation.^31^ Indeed, our study includes a social media–active cohort. However, our study emphasizes previous reports in the literature that dermatologists express fears regarding professional boundaries, accidentally sharing incorrect information or becoming the subject of scrutiny.^32^ On the other side of the social media divide, patients increasingly turn to online communities of practice rather than clinicians, entering potentially harmful and inaccurate echo chambers due to mistrust of professionals.^33–35^ Despite the risks of social media perpetuating disinformation, online platforms could provide patients with an informal support network for those who might be feeling dismissed by HCPs.

Mapping the doctor–patient divide can help synthesize recommendations for practice and research. Practically, a communication toolkit for challenging social media–driven narratives has been developed and could be used in a clinical setting.^36^ However, steps such as training dermatologists on social media literacy, development of innovative education materials and calling for account verification could be key in reaching out to patients.^37^ Reflection of the role of unconscious bias within clinical reasoning could also be implemented during dermatology training to minimize potential negative attitudes. Meaningful engagement with patient leaders in relevant organizations such as ITSAN or Scratch That may demonstrate that dermatologists are willing to listen, and to co-create best practice guidelines. When concerning specific management steps, Brookes *et al.* identified that 26% of their TSW patients experienced comorbid anxiety and depression.^4^ This reinforces the benefits of referring to relevant psychodermatology or psychology services (a treatment option chosen by 22.3% of clinicians in this study). Indeed, dermatologists providing care for patients with self-diagnosed TSW must take a holistic approach and address patient’s psychological wellbeing and how they are coping with not only their skin disease, but also the experience of TSW, in order to improve treatment outcomes, engagement with healthcare and trust in the system.

Finally, this research further suggests that study of the lived experience of TSW patients on social media should be considered urgently, perhaps using qualitative approaches such as digital ethnography.^38^ Future research amongst other healthcare practitioner populations would likely be of value, including general practitioners or dermatology specialist nurses.

Limitations of this study include the response rate, which may be considered low; however, it is comparable to previous similar studies. Responses outside of England are likely to be underrepresented. Furthermore, whilst use of a control arm was intended to introduce relevant comparison, it is possible that consent and information materials may have hinted at TSW being a feature of the study, influencing results. To mitigate against this, participants were informed that they were taking part in research on ‘conditions relating to topical steroids’. Whilst adapting established questionnaires used in similar historical settings should be considered a strength, survey response bias may exist due to framing of questions on clinician attitudes.

TSW is dermatology’s first truly patient-led, social-­media driven phenomenon. This study helps map the divide between patients and dermatologists on TSW attitudes and provides a snapshot of current concerns, practice and experiences of dermatologists related to TSW. Becoming cognizant of subconscious negative attitudes, practising compassionate communication, and co-creating of education and research in a collaborative social media climate may prove crucial in bridging this important gap.

## Data Availability

The data that support the findings of this study are available from the corresponding author, J.G., upon reasonable request.
