# Correction to: Topical steroid withdrawal: self-diagnosis, unconscious bias and social media

**DOI:** 10.1093/skinhd/vzaf064

**Published:** 2025-07-29

This is a correction to: Jonathan Guckian, Olivia Hughes, Yasmin Nikookam, Ria Nair, Aqua Asif, Jeremy Brown, Anthony Bewley, Faheem Latheef, Topical steroid withdrawal: self-diagnosis, unconscious bias and social media, Skin Health and Disease, 2025, https://doi.org/10.1093/skinhd/vzaf051

The email address for the correspondence author should read j.guckian@leeds.ac.uk

The first paragraph of subsection ‘Participant demographics’ in the ‘Results’ section, which reads ‘One hundred and three respondents to the survey met the inclusion criteria for the study including Dermatology Consultants (*n* = 51/103, 49.5%), Dermatology Registrars (*n* = 38/103, 36.9%), Dermatology Fellows (*n* = 10/103, 9.8%), Specialty and Specialist (SAS) Dermatology doctors (*n* = 3/103, 2.9%) and Post-CCT (Certificate of Completion of Training) Fellows (1/103, 1%). The study received responses from across the UK, including England (*n* = 78, 75.7%), Wales (*n* = 6, 5.8%), Scotland (*n* = 11, 10.7%) and Northern Ireland (*n* = 8, 7.7%). Of the participants, 66 were female (64%), 33 were male (32%) and 4 (3.8%) preferred not to disclose their gender’, should read ‘One hundred and three respondents to the survey met the inclusion criteria for the study including Dermatology Consultants (*n* = 51/103, 49.5%), Dermatology Registrars (*n* = 38/103, 36.9%), Dermatology Fellows (*n* = 10/103, 9.7%), Specialty and Specialist (SAS) Dermatology doctors (*n* = 3/103, 2.9%) and Post-CCT (Certificate of Completion of Training) Fellows (*n* = 1/103, 1.0%). The study received responses from across the UK, including England (*n* = 78, 75.7%), Wales (*n* = 6, 5.8%), Scotland (*n* = 11, 10.7%) and Northern Ireland (*n* = 8, 7.8%). Of the participants, 66 were female (64.1%), 33 were male (32.0%) and 4 (3.9%) preferred not to disclose their gender.’

The text in the second paragraph of the subsection ‘Scenario responses’ in the ‘Results’ section, which reads ‘We found that the 46.6% (*n* = 48/103) of respondents who were allocated to the TSW self-diagnosis scenario were significantly more likely to believe that the patient was ‘more likely to post difficult management problems’ (mean score 3.83/5 vs 2.81/5, *P* = 0.001) and ‘more likely to take up one’s time’ (4/5 vs 3.27/5, *P* < 0.001) than the 53.4% (*n* = 55/103) of participants allocated to the patient not self-diagnosing TSW’ should read ‘We found that the 46.6% (*n* = 48/103) of respondents who were allocated to the TSW self-diagnosis scenario were significantly more likely to believe that the patient was ‘more likely to post difficult management problems’ (mean score 3.83/5 vs. 2.91/5, *P* = 0.001) and ‘more likely to take up one’s time’ (4/5 vs. 3.27/5, *P* < 0.001) than the 53.4% (*n* = 55/103) of participants allocated to the group of patients not self-diagnosing TSW.’

The text in the second paragraph of the subsection ‘Perspectives on TSW’ in the ‘Results’ section, which reads ‘Only 18% (*n* = 16/89) participants felt their TSW patients were receiving adequate care, whilst 37.5% (*n* = 33/89) felt that their TSW patients’ care was inadequate and 44.9% (*n* = 40/89) were unsure’, should read ‘Only 18% (*n* = 16/89) of participants felt their TSW patients were receiving adequate care, whilst 37.1% (*n* = 33/89) felt that their TSW patients’ care was inadequate and 44.9% (n = 40/89) were unsure.’

In Table 1, the data were not provided in the clearest format. Table 1 should be presented as follows:

**Table 1:** Clinician attitudes (*n* = 103) toward a self-diagnosis of topical steroid withdrawal (TSW) vs. no self-diagnosis of TSW

	Level of agreement				
	Scenario 1 (*n* = 48): TSW concerns mentioned		Scenario 2 (*n* = 55): no TSW concerns mentioned		
	Mean (SD) score	95% CI	Mean (SD) score	95% CI	*P*-value
This patient is likely to comply with treatment	2.77 (1.06)	2.46–3.07	3.40 (1.04)	3.12–3.68	0.003*
I would not like to have this patients in my clinic	2.69 (1.29)	2.31–3.06	1.89 (0.96)	1.63–2.15	0.001*
This patient poses difficult management problems	3.83 (0.81)	3.60–4.07	2.91 (1.14)	2.60–3.22	< 0.001*
This patient is likely to take up a lot of one’s time	4.00 (0.74)	3.78–4.22	3.27 (1.06)	3.07–3.68	< 0.001*
I would prescribe topical steroids for this patient	3.40 (1.01)	3.34–3.95	3.71 (1.07)	3.42–4.00	0.11

*Significance at *P* < 0.05.

